# Echinocandins: A ray of hope in antifungal drug therapy

**DOI:** 10.4103/0253-7613.71906

**Published:** 2010-12

**Authors:** Neeta D. Grover

**Affiliations:** Department of Pharmacology, Bharati Vidyapeeth University Medical College and Hospital, Sangli, Maharashtra, India

Sir,

I would like to thank the respondents for expressing an interest in the article.[1] They have raised two important issues.

1. Micafungin was approved for pediatric patients, including neonates, for prophylaxis of candidial infections in patients undergoing HSCT and in those with invasive candidiasis in Japan and the European Union. I have shown only the USFDA-approved indications in the study.[2] The drug regulatory authority in the USA (USFDA) does not permit the use of micafungin in the pediatric population.[3] However, the European Medicines Agency or EMEA permits the use of micafungin in the pediatric population.[4] As per the latest available information, micafungin is still not approved for pediatric population by the USFDA, due to limited experience. This discrepancy between the USFDA and the EMEA is not a first time occurrence, especially in the area of antimicrobials.[5]
2. As far as its being an alternative to fluconazole, in the case of prophylaxis in the preterm neonates, even the EMEA cautions against the use of micafungin, if an acceptable alternative is available. This is due to a suspected risk of hepatic tumors seen as foci of altered hepatocytes (FAH) in animal studies, which did not get reversed on stopping the drug, but developed into tumors. Moreover, the incidence of derangement of hepatic transaminases was significantly higher in the pediatric age group in clinical trials. The hepatic adverse events are highest in the below 1 year age group.[4,6] While granting marketing authorization, the Committee for Human Medicinal Products (CHMP) concluded that due to a potential risk of the development of hepatic tumors, the benefit/risk ratio of all other applicable antifungals is considered “superior” to micafungin.[7] It may, of course, be a matter of time before the drug is approved for use in the pediatric population in the United States.
